# μ-Aqua-κ^2^ *O*:*O*-di-μ-4-methyl­benzoato-κ^4^ *O*:*O*′-bis­[(4-methyl­benzoato-κ*O*)(1,10-phenanthroline-κ^2^ *N*,*N*′)nickel(II)]

**DOI:** 10.1107/S1600536808017285

**Published:** 2008-06-13

**Authors:** Wen-Dong Song, Jian-Bin Yan, Xiao-Min Hao

**Affiliations:** a College of Science, Guang Dong Ocean University, Zhan Jiang 524088, People’s Republic of China

## Abstract

In the title dinuclear complex, [Ni_2_(C_8_H_7_O_2_)_4_(C_12_H_8_N_2_)_2_(H_2_O)], each Ni^II^ atom is six-coordinated by three carboxylate O atoms from three 4-methyl­benzoate ligands, two N atoms from two 1,10-phenanthroline ligands, and one μ_2_-bridging aqua ligand. The dimeric complex is located on a crystallographic twofold axis and each Ni atom displays a distorted octa­hedral coordination geometry. The crystal structure is stabilized *via* intra­molecular hydrogen bonding of the bridging water mol­ecule and the uncoordinated carboxyl­ate O atoms, and by C—H⋯O and π–π stacking inter­actions [centroid–centroid distances between neighbouring phenanthroline ring systems and between the benzene ring of a 4-methyl­benzoate unit and a phenanthroline ring system are 3.662 (2) and 3.611 (3) Å, respectively].

## Related literature

For the coordination chemistry of 4-methylbenzoate complexes see: Song *et al.* (2007 ▶); Li *et al.* (2003 ▶, 2004 ▶); Geetha *et al.* (1999 ▶). For related complexes, see: Eremenko *et al.* (1999 ▶); Sung *et al.* (2000 ▶); Novak *et al.* (2005 ▶).
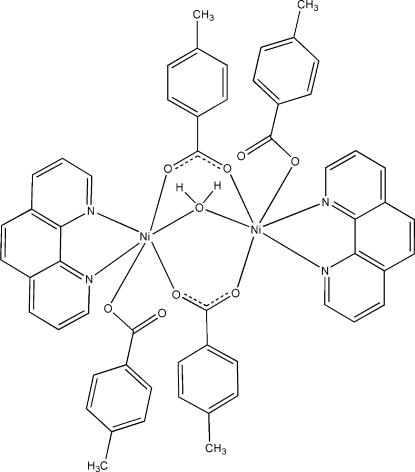


## Experimental

### 

#### Crystal data

[Ni_2_(C_8_H_7_O_2_)_4_(C_12_H_8_N_2_)_2_(H_2_O)]
*M* *_r_* = 1036.39
Monoclinic, 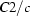

*a* = 23.4180 (6) Å
*b* = 15.4595 (4) Å
*c* = 15.6140 (3) Å
β = 122.351 (1)°
*V* = 4775.4 (2) Å^3^
*Z* = 4
Mo *K*α radiation
μ = 0.85 mm^−1^
*T* = 296 (2) K
0.35 × 0.32 × 0.26 mm

#### Data collection

Bruker APEXII area-detector diffractometer
Absorption correction: multi-scan (*SADABS*; Sheldrick, 1996 ▶) *T*_min_ = 0.612, *T*_max_ = 0.801
23989 measured reflections
5125 independent reflections
3585 reflections with *I* > 2σ(*I*)
*R*_int_ = 0.077

#### Refinement

*R*[*F* ^2^ > 2σ(*F* ^2^)] = 0.043
*wR*(*F* ^2^) = 0.117
*S* = 1.08
5125 reflections
326 parameters
1 restraint
H atoms treated by a mixture of independent and constrained refinement
Δρ_max_ = 0.40 e Å^−3^
Δρ_min_ = −0.49 e Å^−3^

### 

Data collection: *APEX2* (Bruker, 2004 ▶); cell refinement: *SAINT* (Bruker, 2004 ▶); data reduction: *SAINT*; program(s) used to solve structure: *SHELXS97* (Sheldrick, 2008 ▶); program(s) used to refine structure: *SHELXL97* (Sheldrick, 2008 ▶); molecular graphics: *XP* in *SHELXTL*; software used to prepare material for publication: *SHELXTL*.

## Supplementary Material

Crystal structure: contains datablocks I, global. DOI: 10.1107/S1600536808017285/zl2119sup1.cif

Structure factors: contains datablocks I. DOI: 10.1107/S1600536808017285/zl2119Isup2.hkl

Additional supplementary materials:  crystallographic information; 3D view; checkCIF report

## Figures and Tables

**Table 1:** Hydrogen-bond geometry (Å, °)

*D*—H⋯*A*	*D*—H	H⋯*A*	*D*⋯*A*	*D*—H⋯*A*
C1—H1⋯O4^i^	0.93	2.49	3.007 (3)	115
C6—H6⋯O2^ii^	0.93	2.52	3.296 (4)	142
C8—H8⋯O3^iii^	0.93	2.52	3.379 (4)	153
O1*W*—H1*W*⋯O2^i^	0.830 (10)	1.746 (12)	2.560 (2)	166 (3)

Symmetry codes: (i) 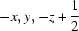; (ii) 
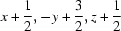
; (iii) 
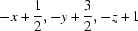
.

**Fig. 1.**
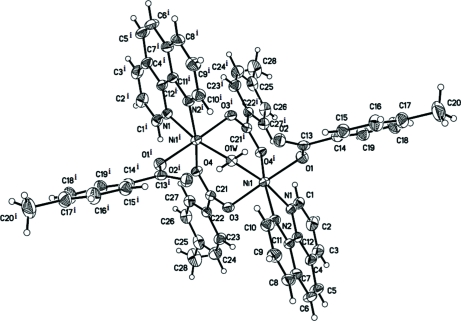
The structure of (I), showing the atomic numbering scheme. Non-H atoms are shown as 30% probability displacement ellipsoids. Symmetry code i = -x, y, -z+1/2.

**Fig. 2.**
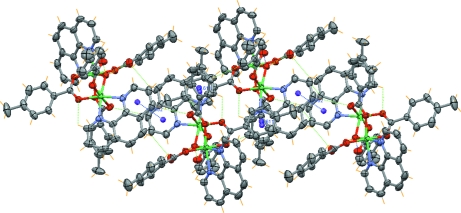
A packing view of the title compound. The purple spheres represent ring centroids involved in π-π stacking interactions (blue dashed lines). The green dashed lines represent C—H···O and O—H···O hydrogen bonds.

## supplementary crystallographic information

### Comment

In the structural investigation of 4-methylbenzoate complexes, it has been found that 4-methylbenzoic acid can function as a multidentate ligand [Song *et al.* (2007); Li *et al.* (2003); Li *et al.* (2004); Geetha *et al.* (1999)], with versatile binding and coordination modes. In this paper, we report the crystal structure of the title compound, (I), a new Ni complex obtained by the reaction of 4-methylbenzoic acid, 1,10-phenanthroline and nickel chloride in alkaline aqueous solution.

As illustrated in Figure 1, each Ni^II^ atom, lies on a crystallographic two fold axis, and has a distorted octahedral geometry with the six coordinating atoms being three carboxyl O atoms from two µ_2_-bridging 4-methylbenzoate ligands and one 4-methylbenzoate ligand, two N atoms from two 1,10-phenanthroline ligands, and one µ_2_-bridging aqua ligand. Therefore, the O1W water molecule bridges both Ni atoms [Ni1···O1W···Ni2^i^ 110.40 (11)°, symmetry code i = -x, y, -z+1/2] and with a Ni···Ni^i^ distance of 3.449 (3) Å. This value is similar to that observed for a binuclear pivalate complexes with a bridging water molecule Ni_2_L_4_(µ-OH_2_)(µ-OOCCMe_3_)_2_(OOCCMe_3_)_2_, (L_2_=Py_2_, (3,4-lutidine)_2_, (N-nitroxyethylnicotinamide)_2_, Dipy) [Eremenko *et al.* (1999)], for which ferromagnetic spin exchange was observed. The Ni···O1W distance is 2.100 (14) Å which is a little shorter than that in other similar complexes [Sung *et al.*,  2000; Novak *et al.*, 2005], suggesting their non-negligible interactions.

The interactions of the structural components are governed by O—H···O hydrogen bonds, C—H···O interactions (Table 1) and by two types of π-π stacking interactions between two closeby phenantroline rings and between a phenyl ring of a 4-methylbenzoate unit and a phenantroline unit. The centroid to centroid distances for the further π-π stacking interaction is 3.662 (2) Å [symmetry code = x, -y, z-1/2], that of the latter 3.611 (3) Å [symmetry code = 1/2-x, 1/2-y, 1-z], respectively, thus indicating weak π-π stacking interactions (Fig. 2).

### Experimental

A mixture of nickel chloride (1 mmol), 4-methylbenzate (1 mmol), 1,10-phenanthroline (1 mmol), NaOH (1.5 mmol) and H_2_O (12 ml) was placed in a 23 ml Teflon reactor, which was heated to 433 K for three days and then cooled to room temperature at a rate of 10 K h^-1^. The crystals obtained were washed with water and dryed in air.

### Refinement

Carbon-bound H atoms were placed at calculated positions and were treated as riding on the parent C atoms with C—H = 0.93–0.97 Å, and with *U*_iso_(H) = 1.2 *U*_eq_(C). Water H atoms were tentatively located in difference Fourier maps and were refined with distance restraints of O–H = 0.82 Å, each within a standard deviation of 0.01 Å with *U*_iso_(H) = 1.5 *U*_eq_(O).

### Crystal data

**Table:**

[Ni_2_(C_8_H_7_O_2_)_4_(C_12_H_8_N_2_)_2_(H_2_O)]	*Z* = 4
*M**_r_* = 1036.39	*F*_000_ = 2152
Monoclinic, *C*2/*c*	*D*_x_ = 1.442 Mg m^−^^3^
Hall symbol: -C 2yc	Mo *K*α radiation λ = 0.71073 Å
*a* = 23.4180 (6) Å	θ = 1.3–28.0º
*b* = 15.4595 (4) Å	µ = 0.85 mm^−^^1^
*c* = 15.6140 (3) Å	*T* = 296 (2) K
β = 122.3510 (10)º	Block, blue
*V* = 4775.4 (2) Å^3^	0.35 × 0.32 × 0.26 mm

### Data collection

**Table:**

Bruker APEXII area-detector diffractometer	5125 independent reflections
Radiation source: fine-focus sealed tube	3585 reflections with *I* > 2σ(*I*)
Monochromator: graphite	*R*_int_ = 0.077
*T* = 296(2) K	θ_max_ = 27.0º
φ and ω scans	θ_min_ = 1.7º
Absorption correction: multi-scan(SADABS; Sheldrick, 1996)	*h* = −29→29
*T*_min_ = 0.612, *T*_max_ = 0.801	*k* = −19→18
23989 measured reflections	*l* = −19→19

### Refinement

**Table:**

Refinement on *F*^2^	Secondary atom site location: difference Fourier map
Least-squares matrix: full	Hydrogen site location: inferred from neighbouring sites
*R*[*F*^2^ > 2σ(*F*^2^)] = 0.043	H atoms treated by a mixture of independent and constrained refinement
*wR*(*F*^2^) = 0.117	*w* = 1/[σ^2^(*F*_o_^2^) + (0.0505*P*)^2^ + 0.0814*P*] where *P* = (*F*_o_^2^ + 2*F*_c_^2^)/3
*S* = 1.08	(Δ/σ)_max_ = 0.001
5125 reflections	Δρ_max_ = 0.40 e Å^−^^3^
326 parameters	Δρ_min_ = −0.49 e Å^−^^3^
1 restraint	Extinction correction: none
Primary atom site location: structure-invariant direct methods	

### Special details

**Table:**

Geometry. All esds (except the esd in the dihedral angle between two l.s. planes) are estimated using the full covariance matrix. The cell esds are taken into account individually in the estimation of esds in distances, angles and torsion angles; correlations between esds in cell parameters are only used when they are defined by crystal symmetry. An approximate (isotropic) treatment of cell esds is used for estimating esds involving l.s. planes.
Refinement. Refinement of F^2^ against ALL reflections. The weighted R-factor wR and goodness of fit S are based on F^2^, conventional R-factors R are based on F, with F set to zero for negative F^2^. The threshold expression of F^2^ > 2sigma(F^2^) is used only for calculating R-factors(gt) etc. and is not relevant to the choice of reflections for refinement. R-factors based on F^2^ are statistically about twice as large as those based on F, and R- factors based on ALL data will be even larger.

### Fractional atomic coordinates and isotropic or equivalent isotropic displacement parameters (Å^2^)

**Table:**

	*x*	*y*	*z*	*U*_iso_*/*U*_eq_	
Ni1	0.045434 (16)	0.85690 (2)	0.38073 (2)	0.03319 (13)	
C1	0.09421 (15)	1.00994 (18)	0.5312 (2)	0.0456 (7)	
H1	0.0520	1.0348	0.4882	0.055*	
C2	0.14290 (18)	1.0573 (2)	0.6149 (2)	0.0573 (8)	
H2	0.1330	1.1122	0.6277	0.069*	
C3	0.20514 (18)	1.0217 (2)	0.6776 (2)	0.0588 (9)	
H3	0.2383	1.0529	0.7330	0.071*	
C4	0.21914 (15)	0.9386 (2)	0.6588 (2)	0.0505 (8)	
C5	0.28260 (17)	0.8954 (3)	0.7205 (3)	0.0660 (10)	
H5	0.3179	0.9244	0.7757	0.079*	
C6	0.29252 (16)	0.8141 (3)	0.7008 (2)	0.0653 (10)	
H6	0.3343	0.7879	0.7430	0.078*	
C7	0.23985 (14)	0.7668 (2)	0.6159 (2)	0.0491 (8)	
C8	0.24653 (16)	0.6815 (2)	0.5925 (3)	0.0573 (9)	
H8	0.2869	0.6518	0.6331	0.069*	
C9	0.19382 (17)	0.6424 (2)	0.5102 (3)	0.0569 (8)	
H9	0.1973	0.5850	0.4955	0.068*	
C10	0.13391 (15)	0.68918 (19)	0.4475 (2)	0.0480 (7)	
H10	0.0986	0.6623	0.3902	0.058*	
C11	0.17777 (13)	0.80871 (18)	0.55122 (19)	0.0408 (7)	
C12	0.16736 (14)	0.89602 (19)	0.5737 (2)	0.0413 (6)	
C13	−0.03124 (13)	0.72317 (18)	0.4141 (2)	0.0382 (6)	
C14	−0.04880 (13)	0.68008 (18)	0.4834 (2)	0.0400 (6)	
C15	−0.05758 (15)	0.59133 (19)	0.4793 (2)	0.0492 (7)	
H15	−0.0538	0.5590	0.4324	0.059*	
C16	−0.07204 (18)	0.5503 (2)	0.5444 (3)	0.0593 (9)	
H16	−0.0767	0.4904	0.5415	0.071*	
C17	−0.07961 (17)	0.5960 (2)	0.6133 (3)	0.0608 (9)	
C18	−0.07257 (18)	0.6851 (2)	0.6154 (3)	0.0650 (9)	
H18	−0.0786	0.7177	0.6601	0.078*	
C19	−0.05673 (16)	0.7264 (2)	0.5519 (2)	0.0526 (8)	
H19	−0.0514	0.7862	0.5555	0.063*	
C20	−0.0955 (2)	0.5511 (3)	0.6843 (3)	0.0894 (13)	
H20A	−0.0833	0.5882	0.7409	0.134*	
H20B	−0.0703	0.4982	0.7082	0.134*	
H20C	−0.1430	0.5385	0.6489	0.134*	
C21	0.07639 (13)	0.96624 (16)	0.2598 (2)	0.0345 (6)	
C22	0.11522 (13)	1.04903 (17)	0.2811 (2)	0.0372 (6)	
C23	0.17742 (15)	1.0578 (2)	0.3710 (2)	0.0522 (8)	
H23	0.1939	1.0137	0.4189	0.063*	
C24	0.21486 (18)	1.1324 (2)	0.3892 (3)	0.0652 (10)	
H24	0.2571	1.1369	0.4488	0.078*	
C25	0.19139 (19)	1.1998 (2)	0.3216 (3)	0.0600 (9)	
C26	0.12895 (18)	1.19063 (19)	0.2329 (3)	0.0562 (8)	
H26	0.1119	1.2356	0.1861	0.067*	
C27	0.09145 (15)	1.11616 (18)	0.2125 (2)	0.0446 (7)	
H27	0.0498	1.1112	0.1520	0.054*	
C28	0.2333 (2)	1.2813 (2)	0.3433 (3)	0.0947 (15)	
H28A	0.2293	1.3171	0.3901	0.142*	
H28B	0.2172	1.3124	0.2812	0.142*	
H28C	0.2798	1.2658	0.3722	0.142*	
N1	0.10564 (11)	0.93089 (14)	0.51042 (16)	0.0379 (5)	
N2	0.12593 (11)	0.77022 (14)	0.46695 (16)	0.0385 (5)	
O1	−0.00788 (10)	0.79909 (12)	0.43632 (15)	0.0443 (5)	
O2	−0.04096 (10)	0.68100 (13)	0.33837 (15)	0.0501 (5)	
O3	0.10179 (9)	0.90933 (11)	0.32838 (13)	0.0407 (4)	
O4	0.02143 (9)	0.95844 (11)	0.17574 (13)	0.0382 (4)	
O1W	0.0000	0.77938 (16)	0.2500	0.0367 (6)	
H1W	0.0180 (14)	0.7449 (15)	0.231 (2)	0.055*	

### Atomic displacement parameters (Å^2^)

**Table:**

	*U*^11^	*U*^22^	*U*^33^	*U*^12^	*U*^13^	*U*^23^
Ni1	0.0289 (2)	0.0333 (2)	0.03124 (19)	−0.00013 (14)	0.01199 (15)	0.00146 (14)
C1	0.0499 (18)	0.0420 (17)	0.0396 (15)	−0.0063 (13)	0.0203 (15)	−0.0012 (13)
C2	0.073 (2)	0.0460 (19)	0.0487 (18)	−0.0183 (16)	0.0300 (19)	−0.0083 (15)
C3	0.061 (2)	0.064 (2)	0.0419 (17)	−0.0304 (17)	0.0205 (17)	−0.0091 (15)
C4	0.0402 (17)	0.064 (2)	0.0356 (15)	−0.0185 (15)	0.0125 (14)	0.0014 (14)
C5	0.0388 (19)	0.091 (3)	0.0448 (19)	−0.0174 (18)	0.0070 (16)	0.0044 (18)
C6	0.0310 (17)	0.101 (3)	0.0457 (19)	0.0010 (18)	0.0081 (15)	0.0197 (19)
C7	0.0334 (16)	0.067 (2)	0.0442 (17)	0.0057 (14)	0.0190 (14)	0.0181 (15)
C8	0.0416 (19)	0.073 (2)	0.058 (2)	0.0215 (16)	0.0272 (17)	0.0271 (18)
C9	0.054 (2)	0.053 (2)	0.064 (2)	0.0190 (16)	0.0329 (19)	0.0184 (16)
C10	0.0445 (18)	0.0455 (18)	0.0514 (18)	0.0048 (14)	0.0239 (15)	0.0067 (14)
C11	0.0308 (15)	0.0531 (18)	0.0350 (15)	0.0006 (12)	0.0152 (13)	0.0114 (12)
C12	0.0330 (16)	0.0520 (17)	0.0339 (14)	−0.0060 (13)	0.0146 (13)	0.0058 (12)
C13	0.0265 (14)	0.0417 (16)	0.0413 (15)	0.0033 (11)	0.0148 (13)	0.0053 (12)
C14	0.0306 (15)	0.0458 (17)	0.0402 (15)	−0.0008 (12)	0.0166 (13)	0.0048 (12)
C15	0.0525 (19)	0.0475 (19)	0.0493 (18)	−0.0021 (14)	0.0282 (16)	0.0022 (14)
C16	0.073 (2)	0.0474 (19)	0.064 (2)	−0.0109 (16)	0.041 (2)	0.0020 (16)
C17	0.066 (2)	0.065 (2)	0.055 (2)	−0.0126 (17)	0.0349 (19)	0.0052 (16)
C18	0.078 (3)	0.073 (2)	0.062 (2)	−0.0128 (19)	0.049 (2)	−0.0112 (18)
C19	0.058 (2)	0.0500 (18)	0.0551 (19)	−0.0080 (15)	0.0339 (17)	−0.0040 (15)
C20	0.113 (4)	0.099 (3)	0.085 (3)	−0.023 (3)	0.072 (3)	0.006 (2)
C21	0.0323 (15)	0.0353 (15)	0.0360 (14)	−0.0004 (11)	0.0183 (13)	−0.0004 (11)
C22	0.0370 (15)	0.0387 (15)	0.0371 (14)	−0.0039 (12)	0.0206 (13)	−0.0061 (12)
C23	0.0476 (19)	0.0549 (19)	0.0452 (17)	−0.0100 (15)	0.0189 (15)	−0.0063 (14)
C24	0.057 (2)	0.076 (3)	0.054 (2)	−0.0284 (18)	0.0244 (18)	−0.0272 (18)
C25	0.080 (3)	0.051 (2)	0.070 (2)	−0.0269 (17)	0.053 (2)	−0.0229 (17)
C26	0.079 (2)	0.0380 (17)	0.064 (2)	−0.0070 (16)	0.047 (2)	−0.0035 (15)
C27	0.0492 (18)	0.0381 (15)	0.0465 (16)	−0.0061 (13)	0.0256 (15)	−0.0052 (13)
C28	0.128 (4)	0.072 (3)	0.118 (3)	−0.058 (3)	0.088 (3)	−0.047 (2)
N1	0.0359 (13)	0.0400 (13)	0.0321 (11)	−0.0037 (10)	0.0144 (10)	0.0031 (10)
N2	0.0322 (13)	0.0413 (13)	0.0379 (12)	0.0017 (10)	0.0160 (11)	0.0087 (10)
O1	0.0481 (12)	0.0377 (11)	0.0510 (11)	−0.0047 (9)	0.0292 (10)	−0.0003 (9)
O2	0.0545 (13)	0.0541 (13)	0.0440 (11)	−0.0156 (10)	0.0280 (11)	−0.0065 (10)
O3	0.0312 (10)	0.0435 (11)	0.0417 (11)	0.0000 (8)	0.0156 (9)	0.0100 (9)
O4	0.0334 (10)	0.0377 (10)	0.0333 (10)	−0.0050 (8)	0.0110 (9)	−0.0003 (8)
O1W	0.0379 (16)	0.0344 (15)	0.0364 (14)	0.000	0.0190 (13)	0.000

### Geometric parameters (Å, °)

**Table:**

Ni1—O4^i^	2.0533 (17)	C14—C15	1.384 (4)
Ni1—O3	2.0546 (17)	C15—C16	1.386 (4)
Ni1—O1	2.0665 (18)	C15—H15	0.9300
Ni1—N1	2.084 (2)	C16—C17	1.375 (4)
Ni1—O1W	2.1001 (14)	C16—H16	0.9300
Ni1—N2	2.108 (2)	C17—C18	1.386 (5)
C1—N1	1.328 (3)	C17—C20	1.513 (4)
C1—C2	1.396 (4)	C18—C19	1.387 (4)
C1—H1	0.9300	C18—H18	0.9300
C2—C3	1.363 (5)	C19—H19	0.9300
C2—H2	0.9300	C20—H20A	0.9600
C3—C4	1.395 (4)	C20—H20B	0.9600
C3—H3	0.9300	C20—H20C	0.9600
C4—C12	1.395 (4)	C21—O4	1.259 (3)
C4—C5	1.433 (5)	C21—O3	1.262 (3)
C5—C6	1.343 (5)	C21—C22	1.501 (3)
C5—H5	0.9300	C22—C27	1.377 (4)
C6—C7	1.437 (4)	C22—C23	1.386 (4)
C6—H6	0.9300	C23—C24	1.382 (4)
C7—C8	1.399 (4)	C23—H23	0.9300
C7—C11	1.408 (4)	C24—C25	1.371 (5)
C8—C9	1.357 (5)	C24—H24	0.9300
C8—H8	0.9300	C25—C26	1.382 (5)
C9—C10	1.408 (4)	C25—C28	1.520 (4)
C9—H9	0.9300	C26—C27	1.378 (4)
C10—N2	1.326 (3)	C26—H26	0.9300
C10—H10	0.9300	C27—H27	0.9300
C11—N2	1.360 (3)	C28—H28A	0.9600
C11—C12	1.448 (4)	C28—H28B	0.9600
C12—N1	1.352 (3)	C28—H28C	0.9600
C13—O2	1.260 (3)	O4—Ni1^i^	2.0533 (17)
C13—O1	1.263 (3)	O1W—Ni1^i^	2.1001 (14)
C13—C14	1.504 (4)	O1W—H1W	0.830 (10)
C14—C19	1.379 (4)		
			
O4^i^—Ni1—O3	91.85 (7)	C16—C15—H15	119.7
O4^i^—Ni1—O1	91.01 (7)	C17—C16—C15	121.6 (3)
O3—Ni1—O1	177.14 (7)	C17—C16—H16	119.2
O4^i^—Ni1—N1	87.80 (8)	C15—C16—H16	119.2
O3—Ni1—N1	85.72 (8)	C16—C17—C18	117.7 (3)
O1—Ni1—N1	94.35 (8)	C16—C17—C20	121.5 (3)
O4^i^—Ni1—O1W	98.37 (7)	C18—C17—C20	120.8 (3)
O3—Ni1—O1W	86.43 (6)	C17—C18—C19	121.0 (3)
O1—Ni1—O1W	93.19 (6)	C17—C18—H18	119.5
N1—Ni1—O1W	170.16 (6)	C19—C18—H18	119.5
O4^i^—Ni1—N2	167.39 (8)	C14—C19—C18	120.9 (3)
O3—Ni1—N2	87.68 (8)	C14—C19—H19	119.6
O1—Ni1—N2	89.52 (8)	C18—C19—H19	119.6
N1—Ni1—N2	79.60 (9)	C17—C20—H20A	109.5
O1W—Ni1—N2	94.17 (8)	C17—C20—H20B	109.5
N1—C1—C2	122.7 (3)	H20A—C20—H20B	109.5
N1—C1—H1	118.6	C17—C20—H20C	109.5
C2—C1—H1	118.6	H20A—C20—H20C	109.5
C3—C2—C1	119.0 (3)	H20B—C20—H20C	109.5
C3—C2—H2	120.5	O4—C21—O3	124.9 (2)
C1—C2—H2	120.5	O4—C21—C22	118.2 (2)
C2—C3—C4	120.1 (3)	O3—C21—C22	116.8 (2)
C2—C3—H3	120.0	C27—C22—C23	118.8 (3)
C4—C3—H3	120.0	C27—C22—C21	121.7 (2)
C3—C4—C12	116.9 (3)	C23—C22—C21	119.5 (3)
C3—C4—C5	124.4 (3)	C24—C23—C22	119.8 (3)
C12—C4—C5	118.7 (3)	C24—C23—H23	120.1
C6—C5—C4	121.8 (3)	C22—C23—H23	120.1
C6—C5—H5	119.1	C25—C24—C23	121.8 (3)
C4—C5—H5	119.1	C25—C24—H24	119.1
C5—C6—C7	121.3 (3)	C23—C24—H24	119.1
C5—C6—H6	119.4	C24—C25—C26	117.8 (3)
C7—C6—H6	119.4	C24—C25—C28	120.9 (4)
C8—C7—C11	117.6 (3)	C26—C25—C28	121.3 (4)
C8—C7—C6	124.0 (3)	C27—C26—C25	121.3 (3)
C11—C7—C6	118.4 (3)	C27—C26—H26	119.4
C9—C8—C7	119.7 (3)	C25—C26—H26	119.4
C9—C8—H8	120.2	C22—C27—C26	120.5 (3)
C7—C8—H8	120.2	C22—C27—H27	119.7
C8—C9—C10	119.5 (3)	C26—C27—H27	119.7
C8—C9—H9	120.2	C25—C28—H28A	109.5
C10—C9—H9	120.2	C25—C28—H28B	109.5
N2—C10—C9	122.4 (3)	H28A—C28—H28B	109.5
N2—C10—H10	118.8	C25—C28—H28C	109.5
C9—C10—H10	118.8	H28A—C28—H28C	109.5
N2—C11—C7	122.5 (3)	H28B—C28—H28C	109.5
N2—C11—C12	117.6 (2)	C1—N1—C12	117.7 (2)
C7—C11—C12	119.9 (3)	C1—N1—Ni1	128.51 (19)
N1—C12—C4	123.5 (3)	C12—N1—Ni1	113.21 (18)
N1—C12—C11	116.6 (2)	C10—N2—C11	118.2 (2)
C4—C12—C11	119.9 (3)	C10—N2—Ni1	129.91 (19)
O2—C13—O1	124.9 (2)	C11—N2—Ni1	111.72 (18)
O2—C13—C14	117.7 (2)	C13—O1—Ni1	123.86 (17)
O1—C13—C14	117.4 (2)	C21—O3—Ni1	120.08 (16)
C19—C14—C15	118.2 (3)	C21—O4—Ni1^i^	129.80 (16)
C19—C14—C13	122.0 (3)	Ni1—O1W—Ni1^i^	110.41 (11)
C15—C14—C13	119.8 (3)	Ni1—O1W—H1W	129 (2)
C14—C15—C16	120.5 (3)	Ni1^i^—O1W—H1W	96 (2)
C14—C15—H15	119.7		

Symmetry codes: (i) −*x*, *y*, −*z*+1/2.

### Hydrogen-bond geometry (Å, °)

**Table:**

*D*—H···*A*	*D*—H	H···*A*	*D*···*A*	*D*—H···*A*
C1—H1···O4^i^	0.93	2.49	3.007 (3)	115
C6—H6···O2^ii^	0.93	2.52	3.296 (4)	142
C8—H8···O3^iii^	0.93	2.52	3.379 (4)	153
O1W—H1W···O2^i^	0.830 (10)	1.746 (12)	2.560 (2)	166 (3)

Symmetry codes: (i) −*x*, *y*, −*z*+1/2; (ii) *x*+1/2, −*y*+3/2, *z*+1/2; (iii) −*x*+1/2, −*y*+3/2, −*z*+1.
